# Immunopotentiation by Natural Extracts: Mechanisms and Applications in Influenza Vaccine Adjuvant Development

**DOI:** 10.1002/fsn3.71840

**Published:** 2026-05-23

**Authors:** Thi Len Ho, Eun‐Ju Ko

**Affiliations:** ^1^ Interdisciplinary Graduate Program in Advanced Convergence Technology & Science Jeju National University Jeju Republic of Korea; ^2^ Department of Veterinary Medicine, College of Veterinary Medicine Jeju National University Jeju Republic of Korea; ^3^ Veterinary Medical Research Institute Jeju National University Jeju Republic of Korea

**Keywords:** immunomodulation, influenza vaccine, natural adjuvants, natural extracts

## Abstract

Despite the widespread use of seasonal influenza vaccines, their efficacy remains limited particularly among the elderly, immunocompromised individuals, and in the face of antigenically drifted or shifted viral strains. Traditional adjuvants enhance vaccine efficacy but may have limitations related to safety, tolerability, or restricted immune activation. Natural extracts derived from plants, marine organisms, fungi, and algae are gaining attention as novel adjuvants due to their ability to modulate both innate and adaptive immunity with low toxicity. This review highlights key natural compounds such as polysaccharides (fucoidan, chitosan, Advax), saponins (QS‐21, Matrix‐M), and flavonoids (naringenin) and their mechanisms of action, including activation of dendritic cells, cytokine induction, and enhancement of T and B cell responses. These extracts promote balanced Th1/Th2 polarization, memory cell formation, and improved mucosal and systemic immunity. We also summarize in vitro and in vivo methods used for adjuvant screening, including immune cell activation assays, T/B cell co‐culture systems, and murine vaccination models. Natural extract‐based adjuvants offer formulation flexibility for various vaccine platforms and delivery routes, including intranasal and intramuscular administration. Their favorable safety profiles, immunostimulatory potency, and capacity to enhance cross‐protective immunity position them as strong candidates for next‐generation adjuvants for influenza vaccines. Ongoing research may enable their broader application in improving vaccine performance across diverse populations.

## Graphical abstract

Natural Extracts as Candidate Influenza Vaccine Adjuvants. Seasonal influenza vaccines face limitations due to viral mutations and low efficacy in vulnerable populations. Traditional adjuvants often have safety or immune‐activation constraints, leading to increased interest in natural extracts from plants, marine organisms, and fungi. These bioactive compounds, such as saponins and polysaccharides, modulate both innate and adaptive immunity with low toxicity. By activating antigen‐presenting cells (APCs) such as dendritic cells, macrophages, or natural killer (NK) cells and promoting balanced Th1/Th2 responses, they enhance cross‐protective immunity and offer flexible delivery routes. Consequently, natural extracts are promising candidates for next‐generation influenza vaccine adjuvants.

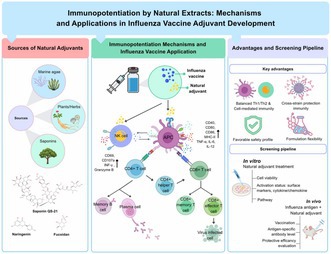

## 1 Introduction

Influenza viruses are highly contagious respiratory pathogens responsible for seasonal epidemics and occasional pandemics worldwide (Treanor 2023). They belong to the Orthomyxoviridae family and are classified into types A, B, and C, with influenza A and B being the primary causes of human disease (Martins et al. 2025; Kim et al. 2022). Influenza A viruses, in particular, are notorious for their ability to undergo antigenic drift and shift, leading to the emergence of new strains that can evade pre‐existing immunity (Maurer et al. 2025; Javanian et al. 2021). This rapid evolution poses significant challenges to public health efforts aimed at controlling the spread of the virus.

Vaccination remains the most effective strategy for preventing influenza infection and mitigating its impact on public health (Taaffe et al. 2024; Hussain 2019). However, the efficacy of current influenza vaccines is suboptimal, especially among vulnerable populations such as the elderly and immunocompromised individuals (Domínguez et al. 2016; Pollard and Bijker 2021). Factors contributing to this limited effectiveness include the antigenic variability of the virus, poor immunogenicity of some vaccine formulations, and waning immunity over time (Xu et al. 2023). These challenges underscore the need for novel approaches to enhance vaccine‐induced protection. To address these limitations, strategies such as the use of adjuvanted vaccines, high‐dose formulations, and alternative delivery platforms have been developed to improve immune responses and broaden protection in high‐risk populations (Caldera et al. 2021; Isakova‐Sivak et al. 2021).

In recent years, there has been increasing interest in the development of novel adjuvants and immune modulators aimed at enhancing the efficacy of influenza vaccines. Among these, natural extracts derived from a wide range of biological sources including medicinal plants, marine algae, fungi, and microbial metabolites have gained attention for their broad immunomodulatory capabilities (Pifferi et al. 2021; Kumar et al. 2022; Gao and Guo 2023). These substances can stimulate both innate and adaptive immune responses by activating antigen‐presenting cells, enhancing cytokine production, and modulating T and B cell functions (Woods, Niwasabutra, Acevedo, et al. 2017; Manilal et al. 2010; Spolaore et al. 2006). Notably, many natural extracts exhibit favorable safety profiles and multifunctional immunological properties, making them especially attractive for use in populations with compromised immunity. Due to their dual ability to enhance immunogenicity while maintaining safety, natural extracts offer a potential pathway to improve the efficacy and durability of influenza vaccines.

## 2 Influenza Vaccines and Their Adjuvants

### 2.1 Influenza Vaccines

Influenza is a recurring global health concern, causing seasonal epidemics that lead to significant morbidity, mortality, and economic burden (Kumari et al. 2023). Globally, seasonal influenza causes roughly 3 to 5 million severe cases and 650,000 deaths annually (WHO 2025). Beyond these seasonal patterns, the recent expansion of Highly Pathogenic Avian Influenza (HPAI) H5N1 has shifted the epidemiological landscape, evolving since 2020 into a persistent panzootic with increasing spillover into mammalian species, including cattle and humans (Krammer et al. 2025). Vaccination remains the most effective method for preventing influenza infection and controlling its spread. Modern influenza vaccines are designed to stimulate the immune system to recognize and respond rapidly to the virus upon exposure, reducing disease severity and transmission.

Several types of influenza vaccines are currently licensed and used globally, each differing in their formulation, mechanism of action, and target population. These vaccines are broadly categorized based on their composition, method of production, and route of administration, and they are tailored to meet the needs of different population groups, including children, the elderly, and immunocompromised individuals (Table 1).

**TABLE 1:** Types of influenza vaccines.

Vaccine type	Approval status	Adjuvant status	Formulations	Immune response	Advantages	Disadvantages/Considerations	References
Inactivated Influenza Vaccines (IIVs)	Licensed	Generally None; but used in Fluad (MF59)	Chemically inactivated virus particles (whole‐virus, split‐virus, or subunit)	Th2‐biased humoral immunity, strain‐specific neutralizing antibodies	Widely used, improved safety with split‐virus and subunit forms	Limited cellular/mucosal immunity, effectiveness wanes over time, higher reactogenicity with whole‐virus	(FDA Ca 2026; Grohskopf et al. 2024; Sano et al. 2018; Chung et al. 2019; Cox et al. 2004; Sridhar et al. 2015)
Live Attenuated Influenza Vaccines (LAIVs)	Licensed	None	Weakened, cold‐adapted, temperature‐sensitive live influenza viruses	Mucosal (IgA) and systemic immunity, Th1 responses, cytotoxic CD8^+^ T cells	Mimics natural infection, robust and broad immune activation, cross‐protective immunity	Contraindicated in very young children, pregnant women, and immunocompromised individuals	(CDC 2022; FDA 2024; Perego et al. 2021; Isakova‐Sivak et al. 2020; Isakova‐Sivak and Rudenko 2015)
Recombinant Influenza Vaccines (RIVs)	Licensed	None	Typically express HA protein in insect cell lines via baculovirus vectors	Strong antibody responses	Avoids egg adaptation, faster production, precise antigenic match, useful for egg allergies/pandemics	Limited cellular response; newer platform	(2025–2026 Flu Season March 2025.”, n.d.; Recombinant Influenza (Flu) Vaccine.” 2024; Carascal et al. 2022; O Murchu et al. 2023)
Virus‐Like Particle (VLP) Vaccines	Preclinical/Phase 1–3	Testing	Structurally mimic native influenza viruses without genetic material	Potent B and T cell stimulation	Safe, potent B and T cell stimulation, potential for broader protection	Emerging platform, still in development	(Immunogenicity of a Quadrivalent Virus‐Like Particles (VLP) Influenza Vaccine in Healthy Adults.” 2020; Kang et al. 2009; Kang et al. 2012; Galloway et al. 2013; Al‐Halifa et al. 2019)
Nanoparticle‐Based Vaccines	Preclinical/Phase 3	Matrix‐M1	Engineered protein particles displaying viral antigens	Implied to improve antigen stability and immune visibility, leading to better responses	Improved antigen stability, immune visibility, potential for broader protection	Emerging platform, still in development	(Phase 3 Pivotal Trial of NanoFlu in Older Adults.” 2023; Sia et al. 2020; Zhang et al. 2026)
Universal Influenza Vaccines	Preclinical/Early Clinical	Novel Adjuvants testing	Focus on conserved viral components (HA stalk domain, M2e, NP, M1) and (Various platforms: recombinant proteins, viral vectors, DNA, mRNA, peptide‐based)	Aims for long‐lasting, broad‐spectrum protection against multiple subtypes/strains	Long‐lasting, broad‐spectrum protection against multiple strains/pandemic variants	Intense research focus, still in preclinical or clinical evaluation	(HHS, NIH Launch Next‐Generation Universal Vaccine Platform for Pandemic‐Prone Viruses.” 2025; Lim et al. 2024; Huang et al. 2023)

The most commonly used are inactivated influenza vaccines (IIVs) (Sano et al. 2018; Chung et al. 2019). These vaccines are composed of virus particles that have been chemically inactivated to prevent replication while retaining immunogenicity. IIVs are further subdivided into whole‐virus, split‐virus, and subunit formulations (Cox et al. 2004; Sridhar et al. 2015). Whole‐virus vaccines contain intact, inactivated virions and were among the earliest vaccine types developed; however, they are associated with higher reactogenicity (Furuya 2012) (Sanders et al. 2014). Split‐virus vaccines, treated with detergents to disrupt viral membranes, offer improved safety (O'Gorman et al. 2014). Subunit vaccines contain only purified viral proteins, typically hemagglutinin (HA) and neuraminidase (NA), and are associated with the lowest risk of adverse effects (Eichelberger and Wan 2014). IIVs are administered intramuscularly and primarily induce a Th2‐biased humoral immune response, leading to the production of strain‐specific neutralizing antibodies (Kim et al. 2020). However, their ability to elicit strong cellular or mucosal immunity is limited, and their effectiveness tends to wane over time.

Live attenuated influenza vaccines (LAIVs) represent another major category and are typically administered intranasally (Perego et al. 2021; Isakova‐Sivak et al. 2020; Isakova‐Sivak and Rudenko 2015). These vaccines contain weakened forms of influenza viruses that are cold‐adapted and temperature‐sensitive, allowing them to replicate in the upper respiratory tract but not in the lower respiratory tract or systemic tissues. LAIVs closely mimic natural infection, leading to robust activation of both the mucosal (IgA) and systemic immune systems, including the induction of Th1 responses and cytotoxic CD8^+^ T cells (Mohn et al. 2020). This broad immune activation makes LAIVs particularly valuable for inducing cross‐protective immunity, although they are contraindicated in very young children, pregnant women, and immunocompromised individuals due to safety concerns.

Recombinant influenza vaccines (RIVs) offer a modern alternative to traditional egg‐ or cell‐based manufacturing methods (Carascal et al. 2022; O Murchu et al. 2023). Using recombinant DNA technology, these vaccines typically express the HA protein in insect cell lines via baculovirus vectors. RIVs are advantageous because they avoid the antigenic drift introduced by egg adaptation, enabling faster production and a more precise antigenic match with circulating strains. These vaccines stimulate strong antibody responses and are particularly useful for individuals with egg allergies or during rapid vaccine deployment in pandemics.

Emerging platforms such as virus‐like particle (VLP) vaccines and nanoparticle‐based vaccines are also gaining attention for their potential to enhance immunogenicity and provide broader protection (Kang et al. 2009, 2012; Galloway et al. 2013; Al‐Halifa et al. 2019). VLPs structurally mimic native influenza viruses without containing genetic material, enabling safe and potent stimulation of both B and T cell responses. Nanoparticle vaccines, such as those being developed by Novavax, use engineered protein particles displaying viral antigens to improve antigen stability and immune visibility (Sia et al. 2020). Both approaches have shown promising results in inducing cross‐reactive antibodies and T cell responses, and several candidates have reached late‐stage clinical trials.

Finally, the development of universal influenza vaccines is an area of intense research focus (Lim et al. 2024; Huang et al. 2023). These vaccines aim to provide long‐lasting, broad‐spectrum protection against multiple influenza subtypes and strains, including pandemic variants. Unlike conventional vaccines that target the highly variable globular head of HA, universal vaccines focus on conserved viral components, such as the HA stalk domain, M2e (extracellular domain of the M2 protein), nucleoprotein (NP), or matrix protein (M1). Platforms for universal vaccines include recombinant proteins, viral vectors, DNA, mRNA, and peptide‐based approaches, with several candidates currently in preclinical or clinical evaluation.

In all cases, the primary goal of influenza vaccination is to elicit protective humoral immunity, particularly through the induction of neutralizing antibodies that target the hemagglutinin (HA) head domain, thereby preventing viral entry into host cells (Taaffe et al. 2024; Isakova‐Sivak et al. 2021; Krammer 2019). In addition to antibody‐mediated protection, cellular immunity, especially the activation of CD4^+^ and CD8^+^ memory T cells, contributes to long‐term immune surveillance and provides cross‐reactive defense against antigenic variants (Janssens et al. 2022).

**FIGURE 1:**
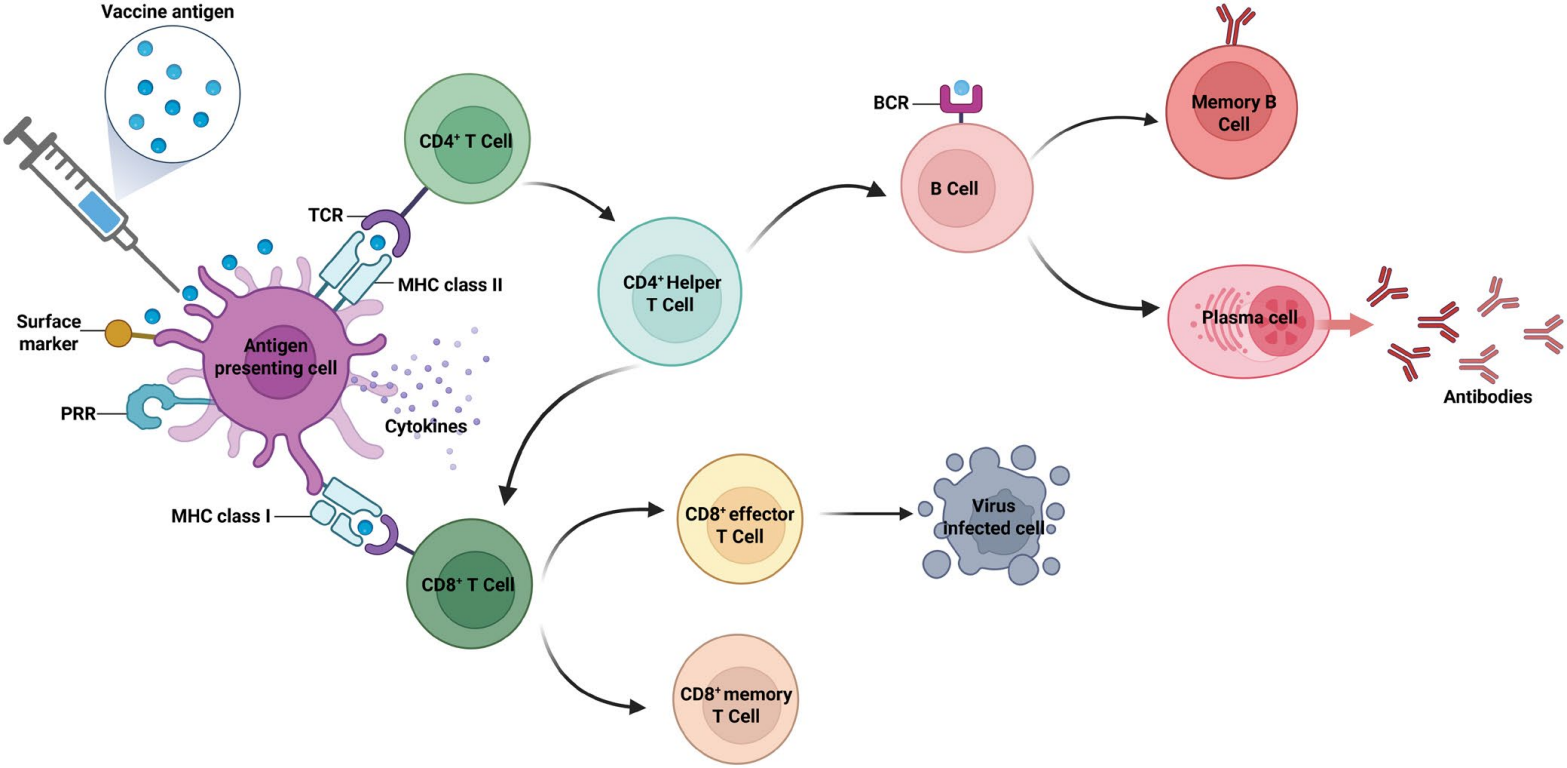
Schematic illustration of immune responses induced by vaccination. The antigen is taken up by antigen‐presenting cells and presented via MHC molecules to T cells. CD4^+^ T cells support B cell activation and antibody production, while CD8^+^ T cells differentiate into cytotoxic and memory cells to eliminate infected cells. Activated B cells give rise to plasma cells and memory B cells, ensuring both immediate and long‐term immunity.

Following vaccine administration, vaccine antigens are recognized and taken up by antigen‐presenting cells (APCs), such as dendritic cells. These cells process the antigens and present antigenic peptides via major histocompatibility complex (MHC) class II molecules to naïve CD4^+^ T cells, promoting their differentiation into helper T cells. These CD4^+^ helper T cells play a central role in coordinating the adaptive immune response by secreting cytokines and providing essential signals for B cell activation. Simultaneously, B cells that encounter the antigen through their surface B cell receptors (BCRs) become activated and, with the support of helper T cells, undergo proliferation, class switching, and differentiation into antibody‐secreting plasma cells and memory B cells. The antibodies produced by plasma cells neutralize circulating viruses, while memory B cells ensure a rapid response upon future exposure. In parallel, dendritic cells also present antigens via MHC class I molecules to activate CD8^+^ T cells. These cells differentiate into cytotoxic T lymphocytes (CTLs), which directly target and eliminate virus‐infected cells. A subset of CD8^+^ T cells further matures into memory T cells, establishing long‐lasting cellular immunity. Together, these pathways coordinate a comprehensive immune response that contributes to both immediate protection and long‐term immunological memory following influenza vaccination (Figure 1).

### 2.2 Adjuvants

Despite their utility, current influenza vaccines have limitations (Table 2), including modest efficacy in certain populations and reduced protection against drifted or mismatched viral strains. These issues stem from antigenic drift and shift (Nypaver et al. 2021), age‐related immune decline, and the focus on variable viral epitopes. These challenges have prompted ongoing efforts to improve vaccine formulations and delivery strategies, including the use of novel adjuvants, alternative platforms, and universal vaccine designs.

**TABLE 2:** Licensed adjuvants used in influenza vaccines.

Adjuvant	Type	Manufacturer	First licensed	Key regulatory status (as of 2026)	Commercial vaccine (s)	Immunological profile	Notes	References
Alum	Aluminum salts	Croda, Sanofi	1924	FDA & EMA Approved	Rarely used in influenza	Strong humoral (Th2); weak cellular immunity	Long history, limited use in influenza due to weak CMI	(Petrovsky and Aguilar 2004; Hogenesch 2013; CDC 2024)
MF59	Squalene‐based oil‐in‐water emulsion	Novartis (now CSL Seqirus)	1997	FDA & EMA Approved	Fluad	Balanced Th1/Th2; enhances antibody and T‐cell responses	Approved for elderly use; well tolerated	(Ko and Kang 2018; O'Hagan et al. 2013; Salk et al. 1951; O'Hagan et al. 2012; Zhang et al. 2025)
AS03	Squalene‐based oil‐in‐water emulsion with α‐tocopherol	GlaxoSmithKline (GSK)	2005 (EU pandemic prep use)	EMA Approved	Pandemrix, Arepanrix	Strong humoral and cellular; slightly Th2‐skewed	Used during H1N1 pandemic; limited use post‐2010	(Garçon et al. 2012; Yang et al. 2013; Hoschler et al. 2014)
AF03	Squalene‐based oil‐in‐water emulsion	Sanofi Pasteur	2009 (clinical use only)	Clinical/Pandemic Use	No current commercial vaccine	Humoral and cellular immunity	Evaluated during H1N1; limited commercial use	(Ustyugova et al. 2023; Caillet et al. 2010; Klucker et al. 2012)
Virosomes	Enveloped Virus‐Like Particle (VLP)	Crucell (now J & J); Berna Biotech; Solvay	1997	EMA Approved (Regional)	*Inflexal V*, *Invivac*	Balanced Th1/Th2 response; triggers both humoral (IgG) and cellular (CTL) immunity	Licensed for all age groups; high safety; not currently FDA‐approved	(Huckriede et al. 2003; Fonseca et al. 2023; Ali et al. 2023; Herzog et al. 2002; Wilschut 2009)

A promising strategy to enhance the efficacy of influenza vaccines, particularly in elderly and immunocompromised populations, is the incorporation of adjuvants (Tregoning et al. 2018). Among the earliest and most extensively used adjuvants are aluminum salts (Alum) known for their safety profile but limited in their ability to induce cellular immunity, mainly promoting Th2‐type responses (Petrovsky and Aguilar 2004; Hogenesch 2013). More advanced adjuvants such as MF59 (Ko and Kang 2018; O'Hagan et al. 2012, 2013; Salk et al. 1951) and AS03 (Garçon et al. 2012), both squalene‐based oil‐in‐water emulsions, have been licensed for use in influenza vaccines like Fluad and Pandemrix, respectively. These adjuvants enhance both humoral and cellular responses, with AS03 showing a tendency to skew toward Th2 polarization while still capable of inducing balanced Th1/Th2 responses. Another emulsion‐based adjuvant, AF03, demonstrated potential in pandemic H1N1 vaccines but has seen limited commercial deployment (Ustyugova et al. 2023; Caillet et al. 2010).

In addition to chemical adjuvants, virosomes represent a highly effective delivery platform that functions as a natural adjuvant by mimicking the structure of a virus. Virosomes consist of reconstituted viral envelopes containing hemagglutinin (HA) and neuraminidase (NA) but lack genetic material (Huckriede et al. 2003; Fonseca et al. 2023; Ali et al. 2023). Unlike many chemical adjuvants, virosomes are uniquely licensed for use in all age groups, including children and infants, due to their excellent safety and tolerability profile (Herzog et al. 2002). They promote a balanced Th1/Th2 immunological profile and are particularly effective at inducing cytotoxic T‐lymphocyte (CTL) responses by delivering antigens directly to the MHC class I pathway (Wilschut 2009).

Although current licensed adjuvants have demonstrated substantial efficacy in enhancing vaccine‐induced immunity, they are associated with certain limitations, including localized reactogenicity and age‐dependent variability in immunogenicity. These constraints have catalyzed the development of next‐generation adjuvants with improved tolerability and broader immune activation profiles. In particular, naturally derived immunostimulatory compounds have emerged as viable candidates due to their capacity to engage both innate and adaptive immune pathways, coupled with favorable biocompatibility and safety characteristics, making them attractive for incorporation into advanced vaccine platforms.

## 3 Natural Extracts as Potential Adjuvant

This review specifically evaluates the potential of natural extracts defined here as bioactive compounds and secondary metabolites derived from plant, fungal, and marine sources to serve as potent influenza vaccine adjuvants (Table 3).

**TABLE 3:** Natural adjuvant components in influenza vaccines.

Compound type	Natural source	Adjuvant features	Application in influenza vaccine	References
Saponins	*Quillaja saponaria* , ginseng	Induce strong Th1 and cytotoxic T cell responses; stimulate DC maturation; used in licensed vaccines (e.g., Matrix‐M, AS01)	Used in AS01 and Matrix‐M systems; improves protection and cross‐reactive responses in seasonal and pandemic influenza vaccines	(Gao and Guo 2023; Silveira et al. 2023; Roman et al. 2024; Bhatnagar et al. 2022; Mbawuike et al. 2007; Nagai et al. 2001; Liu et al. 2012)
Polysaccharides	*Astragalus*, *Ganoderma*, marine algae	Enhance macrophage and dendritic cell activation; stimulate TLR pathways (e.g., TLR4); promote Th1/Th2 cytokine secretion.	Enhances humoral and cellular responses in murine models; reduces required antigen dose (H1N1, H9N2, etc.)	(Zhang et al. 2017; Petrovsky and Cooper 2015; Honda‐Okubo et al. 2012; Khademi et al. 2018; Wan et al. 2024; Zhou et al. 2017; Layton et al. 2011)
Flavonoids/Polyphenols	*Scutellaria, Curcuma longa*, green tea	Exhibit both immunostimulatory and anti‐inflammatory effects; regulate cytokines (IL‐6, TNF‐α, IL‐10); support immune balance	Modulates inflammation and supports vaccine‐induced T and B cell activation; evaluated in experimental influenza adjuvant platforms	(Hannan et al. 2020; Jiao et al. 2025; Yoshino et al. 2009; Fischer et al. 2010)
Delta inulin	Plant‐derived inulin (Advax)	Balanced Th1/Th2 response; increases IgG, IgA, IFN‐γ; well‐tolerated with no significant local reactogenicity	Tested with split H5N1 and other inactivated vaccines; improves survival, antibody titers, and cytokine responses in animal models	(Honda‐Okubo et al. 2012; Pulendran et al. 2021; Honda‐Okubo et al. 2015; Woods, Niwasabutra, Acevedo, et al. 2017; Shinde et al. 2022)

**FIGURE 2:**
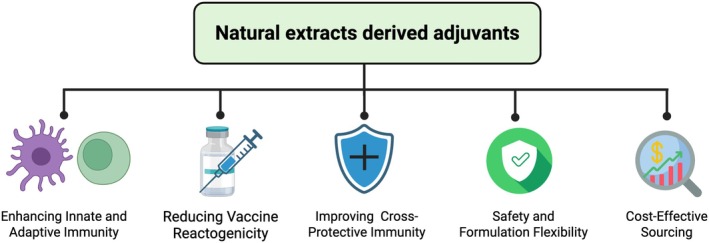
The advantages of natural extract‐derived adjuvants in influenza vaccines. These adjuvants support enhanced innate and adaptive immune activation, reduce adverse inflammatory responses, promote cross‐strain protective immunity, offer flexible and safe formulation options, and enable economically viable sourcing for large‐scale vaccine production.

Natural extracts represent a promising platform for the development of next‐generation vaccine adjuvants, offering a unique balance between immunogenic potency and safety. Beyond their mechanistic appeal, these compounds are increasingly being translated into practical immunological applications aimed at enhancing vaccine efficacy. Sourced from plants, fungi, algae, and other biologically diverse origins, natural extracts are rich in bioactive constituents such as saponins, polysaccharides, and polyphenols that can modulate both innate and adaptive immune responses (Hannan et al. 2020). These compounds exert their effects through a variety of mechanisms, including the activation of antigen‐presenting cells, the promotion of cytokine production, and the regulation of T and B cell function (Pifferi et al. 2021; Kumar et al. 2022; Spolaore et al. 2006; Lin et al. 2020). Their multifunctional immunomodulatory properties, combined with favorable tolerability and biocompatibility, make them particularly attractive candidates for incorporation into both seasonal and pandemic influenza vaccine platforms (Figure 2).

### 3.1 Enhancing Innate and Adaptive Immunity

Natural extracts exert their adjuvant effects through the immunostimulatory activation of antigen‐presenting cells (APCs), including dendritic cells (DCs) and macrophages. These extracts upregulate the expression of major histocompatibility complex (MHC) class I and II molecules, which are essential for antigen processing and presentation to CD8^+^ and CD4^+^ T cells, respectively. They also enhance the expression of co‐stimulatory molecules such as CD40, CD80, and CD86, which provide the secondary signals necessary for full T cell activation and clonal expansion. In addition to surface molecule modulation, natural extracts stimulate the secretion of proinflammatory cytokines (IL‐12, TNF‐α, IL‐6, …), further promoting the maturation and functional capacity of APCs (Manilal et al. 2010; Spolaore et al. 2006).

Marine algal extracts have demonstrated immunostimulatory effects by activating DCs and macrophages, increasing cytokine production (TNF‐α, IL‐6), and upregulating surface markers such as CD40 and CD86 (Ho et al. 2023). *Peyssonnelia caulifera* (PC), a red marine alga, has been shown to activate bone marrow‐derived dendritic cells (BMDCs) and macrophages both in vitro and in vivo via the TLR4 signaling pathway, resulting in increased cytokine secretion and expression of maturation markers (Ho et al. 2024). Another marine‐derived extract, *Cladophora wrightiana* var. *minor* (CW), enhances cellular immunity by activating natural killer (NK) cells and promoting their interactions with dendritic cells (DCs) and CD8^+^ T cells, thereby supporting the efficacy of influenza vaccination (Ho et al. 2025). Similarly, extracts from *Artemisia rupestris L*. promote DC activation and antigen presentation through TLR4‐dependent NF‐κB and MAPK pathways, leading to enhanced expression of MHC‐II and co‐stimulatory molecules (Zhang et al. 2017). When combined with a split influenza vaccine, PC significantly enhanced both Th1 and Th2 responses, increased CD4^+^ and CD8^+^ T cell proliferation in the lungs and spleen, and promoted the formation of memory T and B cells (Ho et al. 2024). Likewise, *Cistanche deserticola* polysaccharide extracts markedly enhanced humoral immune responses, T cell proliferation, and germinal center formation. These effects were associated with increased levels of IFN‐γ in CD4 and CD8 T cells, indicating a balanced Th1/Th2 profile and reduced regulatory T cell (Treg) frequencies (Zhao et al. 2019). In murine models, *Artemisia rupestris L*. extract also increased antigen‐specific IgG1 and IgG2a titers and elevated CD4^+^ and CD8^+^ T cell responses (Zhang et al. 2017).

**FIGURE 3:**
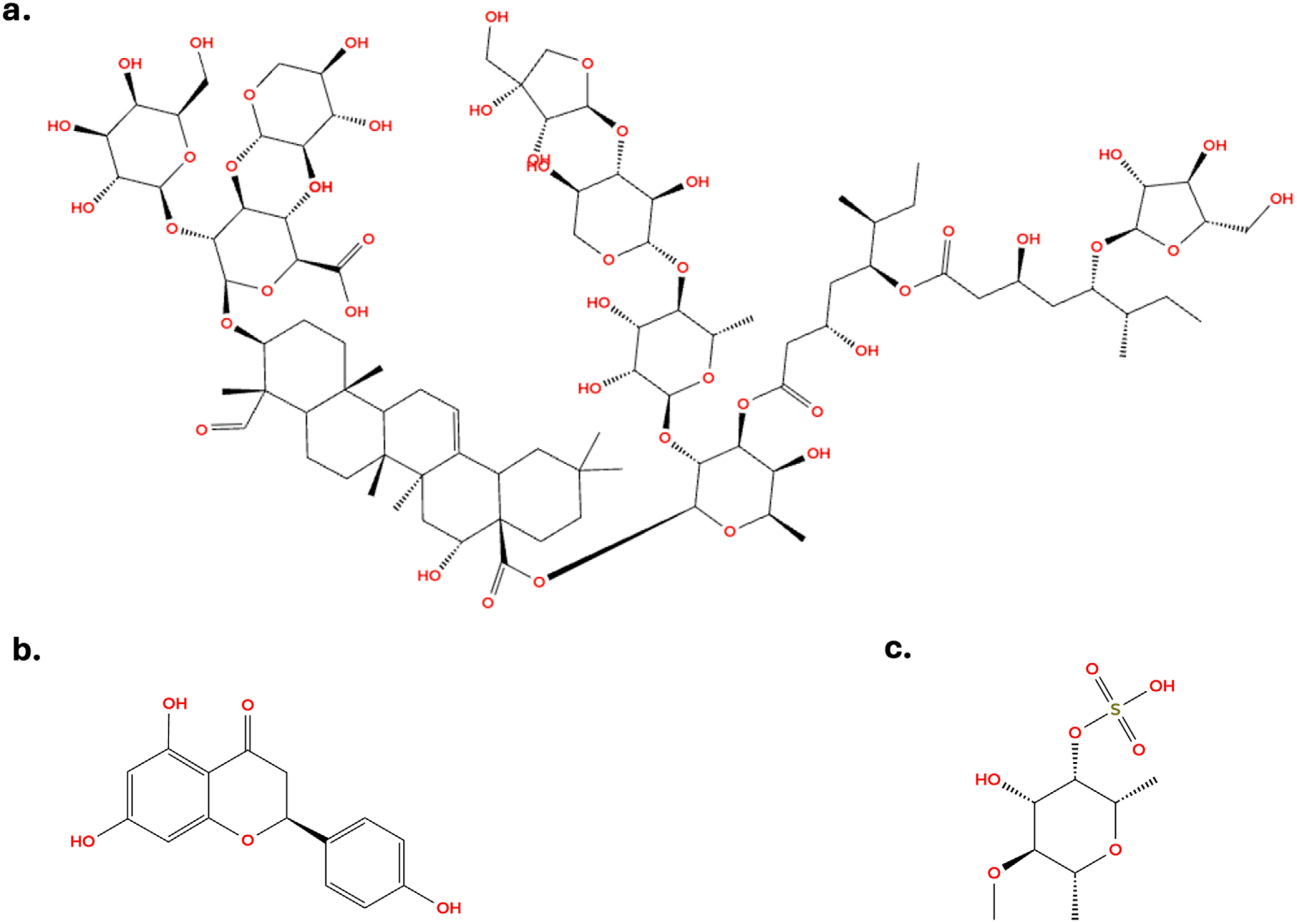
Chemical structures of representative natural adjuvants. (a) Saponin QS‐21: QS‐21 is a triterpene glycoside from *Quillaja saponaria* featuring a quillajic acid nucleus, two carbohydrate chains, and a unique acyl chain. This amphiphilic structure enables cell membrane integration, facilitating antigen cross‐presentation and robust CD8^+^ T‐cell and Th1 responses (PubChem 2026). (b) Flavonoid (Naringenin): Naringenin is a citrus‐derived flavanone with a chromane‐4‐one skeleton and three hydroxyl groups. These phenolic groups provide antioxidant and anti‐inflammatory properties, modulating innate immunity by inhibiting cytokine surges to promote balanced adaptive responses (PubChem NCfBI 2026). (c) Fucoidan: Fucoidan is a sulfated polysaccharide from brown algae composed of alpha‐L‐fucose units. Its high sulfate content and molecular weight are critical for interacting with Pattern Recognition Receptors (e.g., TLR4), triggering dendritic cell maturation to enhance humoral and cellular immunity (Information NCfB 2026).

Saponins (Figure 3a) are glycosides widely found in plants that are well‐known for immunostimulatory properties (Gao and Guo 2023). Quil A, a mixture of saponins from *Quillaja saponaria* bark, and its purified derivative QS‐21 are among the most widely used saponin adjuvants, with effects verified in numerous clinical trials (Silveira et al. 2023). QS‐21 is a purified triterpene glycoside extracted from the bark of *Quillaja saponaria* Molina and is widely recognized for its potent immunostimulatory properties (Lacaille‐Dubois 2019). It is a key component of the AS01 adjuvant system, which is employed in licensed vaccines such as Shingrix (Roman et al. 2024). QS‐21 is known to induce strong Th1‐type immune responses and promote the activation of CD8^+^ cytotoxic T lymphocytes, both of which are essential for effective cellular immunity. When formulated with monophosphoryl lipid A (MPL) in liposomal delivery systems, QS‐21 exhibits enhanced adjuvant effects by stabilizing the saponin structure and targeting delivery to antigen‐presenting cells. In the context of influenza vaccines, the QS‐21 + MPL combination has been shown to significantly amplify both humoral and cellular immune responses including increased levels of IgG2a/c and IFN‐γ producing CD4^+^ T cells and to provide robust protection against both homologous and heterosubtypic influenza virus strains (Bhatnagar et al. 2022) (Mbawuike et al. 2007). Saponin‐adjuvanted influenza vaccines also activate dendritic cells and can even function when CD4 T‐cell help is limited, suggesting a potent direct activation of B cells or innate immune routes (Sun et al. 2014; Zhao et al. 2017). Additional, intranasal administration of saponin‐adjuvanted vaccines has demonstrated protective efficacy against influenza virus infection (Nagai et al. 2001; Liu et al. 2012). *Albizia julibrissin* saponins enhanced immune responses to inactivated H9N2 avian influenza vaccine (IH9V) by rapidly increasing IgG subclasses, HI titers, and IgG levels in mice and chickens. They also promoted splenocyte proliferation, NK cell activity, and balanced Th1 (IL‐2, IFN‐γ) and Th2 (IL‐10) cytokine production with upregulated mRNA expression (Sun et al. 2020).

Fucoidan (Figure 3c), a sulfated polysaccharide derived from the edible seaweed *Undaria pinnatifida* (mekabu), has demonstrated notable immunostimulatory effects in the context of influenza vaccination (Negishi et al. 2013). In a randomized, double‐blind, placebo‐controlled trial involving elderly Japanese individuals, daily oral intake of 300 mg fucoidan for 4 weeks prior to vaccination significantly increased antibody titers against all three seasonal influenza strains (H1N1, H3N2, and B). Moreover, fucoidan supplementation helped maintain or enhance natural killer (NK) cell activity, which otherwise declined in the placebo group over time. Preclinical studies further support these findings (Jin et al. 2014) and showed that fucoidan promotes dendritic cell maturation and drives Th1/Tc1 polarization through IL‐12 signaling. When used alongside ovalbumin antigen, fucoidan significantly enhanced antigen‐specific antibody production and stimulated both CD4^+^ and CD8^+^ T cell proliferation, underscoring its potential as a natural adjuvant for vaccines.

Polysaccharide‐based adjuvants are highly promising due to their ability to engage pattern‐recognition receptors and their excellent safety profiles in influenza vaccination by activating key immune cells and signaling pathways (Petrovsky and Cooper 2015; Honda‐Okubo et al. 2012; Khademi et al. 2018). Many of these polysaccharide adjuvants have demonstrated improved immunogenicity when combined with influenza antigens, including higher serum IgG and mucosal IgA levels. These natural compounds, derived from sources such as *Artemisia rupestris L*., *Cistanche deserticola*, and *Astragalus* (Wan et al. 2024), stimulate antigen‐presenting cells like dendritic cells and macrophages through Toll‐like receptor 4 (TLR4)‐mediated pathways, leading to upregulation of co‐stimulatory molecules (CD40, CD80, CD86), increased cytokine secretion (IL‐6, IL‐12, TNF‐α), and improved antigen presentation (Zhang et al. 2017). Polysaccharides from medicinal plants and fungi such as *Astragalus* root polysaccharides and lentinan from *shiitake* mushrooms also show broad immunopotentiation by activating macrophages, promoting dendritic cell maturation, and enhancing both antibody and T‐cell responses (Zhou et al. 2017). Adjuvants like Advax, a clinically tested microparticle formulation of delta inulin, have been shown to induce both Th1 and Th2 responses, enhancing IgG, IgG2a, IgA, and cytokines such as IFN‐γ and IL‐5, while maintaining excellent safety and stability profiles (Honda‐Okubo et al. 2012). These robust innate immune stimulations support strong adaptive immunity, including enhanced CD4^+^ and CD8^+^ T cell proliferation, balanced Th1/Th2 cytokine responses (IFN‐γ, IL‐4), and elevated production of high‐affinity IgG and mucosal IgA antibodies. Advax with split virion H5N1 vaccine in ferrets demonstrated significantly improved protection against H5N1 virus by increasing immunogenicity, survival, and ultimately morbidity, in comparison to vaccine alone (Layton et al. 2011).

Additionally, chitosan, a polysaccharide derived from crustacean shells, has been widely studied for mucosal delivery, where it increases antigen uptake across nasal mucosa and elicits strong local and systemic immune responses (Ravindranathan et al. 2016; Van der Lubben et al. 2001; Li et al. 2021; Dmour and Islam 2022). Chitosan enhances the efficacy of inactivated influenza vaccines by stimulating both humoral and cellular immunity. It significantly increases hemagglutination‐inhibiting and neutralizing antibody titers, even at lower antigen doses, and induces cross‐protective antibodies against drift. Chitosan activates dendritic cells and macrophages, upregulates MHC I/II and co‐stimulatory molecules, and boosts T cell responses, including increased CD3^+^, CD25^+^, and cytotoxic lymphocyte activity. It also improves splenic cell proliferation and NK activity without inducing IgE or anti‐chitosan antibodies, and retains its adjuvant function after long‐term storage (Ghendon et al. 2008, 2009; Sawaengsak et al. 2014; Mohamed et al. 2018; Dhakal et al. 2018).

Therefore, these findings highlight the ability of natural extracts to modulate APCs function and direct adaptive immune polarization, making them potent candidates for adjuvant development.

### 3.2 Reducing Vaccine Reactogenicity and Safety

The effectiveness of an adjuvant should be balanced with its safety, making it crucial that it enhances immune responses while causing minimal side effects and avoiding excessive inflammation (Hervé et al. 2019; Pulendran et al. 2021). Commonly used adjuvants in licensed human vaccines such as Alum, MF59, AS03, AF03, are generally considered safe and well tolerated. However, despite their strong immunostimulatory properties, some of these adjuvants have been occasionally linked to inflammatory reactions, may increase some local site symptoms, particularly injection site pain including local pain, swelling, pain, fatigue, headache and myalgia granuloma formation, and, in rare cases, systemic effects such as fever or lymphadenopathy (Tregoning et al. 2018; Petrovsky and Aguilar 2004; Aguilar and Rodriguez 2007).

In contrast, some natural product‐based adjuvants offer a more favorable safety profile, as they can activate the immune system without triggering excessive inflammation, thus supporting their potential use in safer vaccine formulations (Pulendran et al. 2021) (Gromer et al. 2024; Lee et al. 2023). Notably, plant‐derived polysaccharides, glycosides, and glycoprotein extracts such as Advax (delta inulin), Matrix‐M, QS‐21, and Mistletoe lectin have been employed as adjuvants in experimental and pandemic‐related vaccines. These agents have consistently demonstrated high immunogenicity while maintaining safety, eliciting balanced humoral and cell‐mediated immune responses across various viral vaccine platforms (Kumar et al. 2022; Honda‐Okubo et al. 2012; Sander et al. 2019; Sette and Crotty 2021; Bisht et al. 2005).

Natural product adjuvants are generally biodegradable substances (polysaccharides or lipids) that do not accumulate or induce long‐term inflammation in tissues. A review of mucosal adjuvants noted that many plant‐derived candidates have inherently low toxicity and minimal side effects in vivo (Petrovsky and Cooper 2015; Honda‐Okubo et al. 2012; Khademi et al. 2018). These include saponins and polysaccharides, which have been safely consumed or used medicinally for centuries (Sun et al. 2009). Because of their biocompatibility and gentle biodegradation, such adjuvants are described as ideal immunomodulators in vaccine research. In traditional medicine contexts, herbs like ginseng or Astragalus (rich in saponins and polysaccharides) are well tolerated; when purified for use in vaccines, these components have similarly high safety margins. For instance, one study emphasizes that the significant immune effects, wide natural availability, and high safety of traditional herbal extracts have led to their testing as vaccine adjuvants.

Evidence from both animal and human studies shows that natural adjuvants can stimulate immunity without provoking strong inflammatory reactions. A clear example is the plant‐derived inulin adjuvant (Advax). In multiple studies, Advax‐adjuvanted vaccines produced no visible sign of inflammation at the injection site and no notable systemic illness in subjects (Honda‐Okubo et al. 2012, 2015; Woods, Niwasabutra, Acevedo, et al. 2017). This contrasts with some oil‐in‐water emulsions or endotoxin‐based adjuvants that often cause injection‐site redness or flu‐like symptoms. In head‐to‐head comparisons, Advax was found to be better tolerated than emulsion adjuvants and did not elicit the fever or local swelling typically seen with alum or MF59. The reason is that Advax does not directly activate inflammatory cytokine pathways—it enhances immune responses through facilitating antigen uptake and presentation rather than by acting as an irritant (Honda‐Okubo et al. 2012; Pulendran et al. 2021; Shinde et al. 2022). Another example, chitosan (polysaccharide) used intranasally, caused no significant nasal irritation in animal models even as it boosted immune responses (Smith et al. 2014).

Similarly, naringenin (Figure 3b), a flavonoid compound, has been reported to upregulate key immune mediators such as IL‐1β, TNF‐α, and the chemokine receptor CCR7, important for lymphocyte trafficking. Interestingly, it also enhances IL‐10 expression, implying a regulatory role in tempering inflammation and supporting immune homeostasis (Jiao et al. 2025).

Overall, natural extract‐based adjuvants offer significant advantages over traditional formulations by avoiding issues such as prolonged local inflammation associated with alum and the elevated reactogenicity of certain synthetic adjuvants. Their favorable safety profiles enhance the overall tolerability of influenza vaccines, which is particularly important for improving vaccine acceptance and enabling safe administration in high‐risk populations, including the elderly, children, and individuals with compromised immunity.

### 3.3 Improving Cross‐Protective Immunity

Regarding influenza vaccines, certain natural adjuvants have demonstrated the ability to enhance cross‐protective immunity. Saponin‐containing adjuvants, such as immunostimulating complexes (ISCOMs), have shown promise (Lövgren Bengtsson et al. 2011; Barr et al. 1998). For instance, ISCOMs were found to significantly increase H1N1 vaccine‐induced hemagglutination inhibition (HI) titer and fully protect mice from heterosubtypic H2N2 viral challenges, with this protection being correlated with cytotoxic T lymphocyte (CTL) responses against a shared major histocompatibility complex (MHC) I epitope within the HA of influenza H1 and H2 viruses (Sambhara et al. 1998). Another saponin‐based adjuvant, Matrix‐M, improved HI titer and protection against drifted H3N2 viruses in ferrets when combined with HA nanoparticles. Matrix‐M also induced cross‐protective immunity against highly pathogenic H5N1 and H7N7 viruses in mice and ferrets when used with a trivalent virosomal vaccine, and in human subjects, the Matrix‐M‐adjuvanted virosomal H5N1 vaccine induced significant microneutralization (MN) antibody titer against heterologous viruses (Cox, Roos, et al. 2015; Cox, Major, et al. 2015).

Enterotoxin adjuvants have also been explored for their ability to enhance cross‐protection. Cholera toxin (CT) significantly increased intranasal (IN) whole‐inactivated PR8 (iPR8) vaccine‐induced HI titer against heterologous and heterosubtypic viruses in murine models, leading to complete protection against heterosubtypic H3N2 viruses, primarily due to cross‐reactive antibodies (Quan et al. 2008). The CT B subunit (CTB) also increased IN trivalent influenza vaccine (TIV)‐induced cross‐protection against a drifted virus in murine models (Tamura et al. 1992). Furthermore, derivatives of heat‐labile enterotoxin (LT), such as LT (R192G), when used with an IN H3N2 vaccine, conferred complete protection against highly pathogenic H5N1 viruses in murine models, with B cells playing a vital role in this heterosubtypic protection (Quan et al. 2008).

### 3.4 Formulation Flexibility

Many natural adjuvants have been used in traditional medicine or as dietary supplements, which supports their safety for human use. Furthermore, they offer formulation flexibility, as they can be incorporated into various vaccine platforms including subunit, inactivated, viral vector, and nanoparticle‐based vaccines. Their compatibility with mucosal delivery routes such as intranasal sprays also opens avenues for non‐invasive immunization strategies.

## 4 Methods for Screening Natural Extracts as Vaccine Adjuvants

**FIGURE 4:**
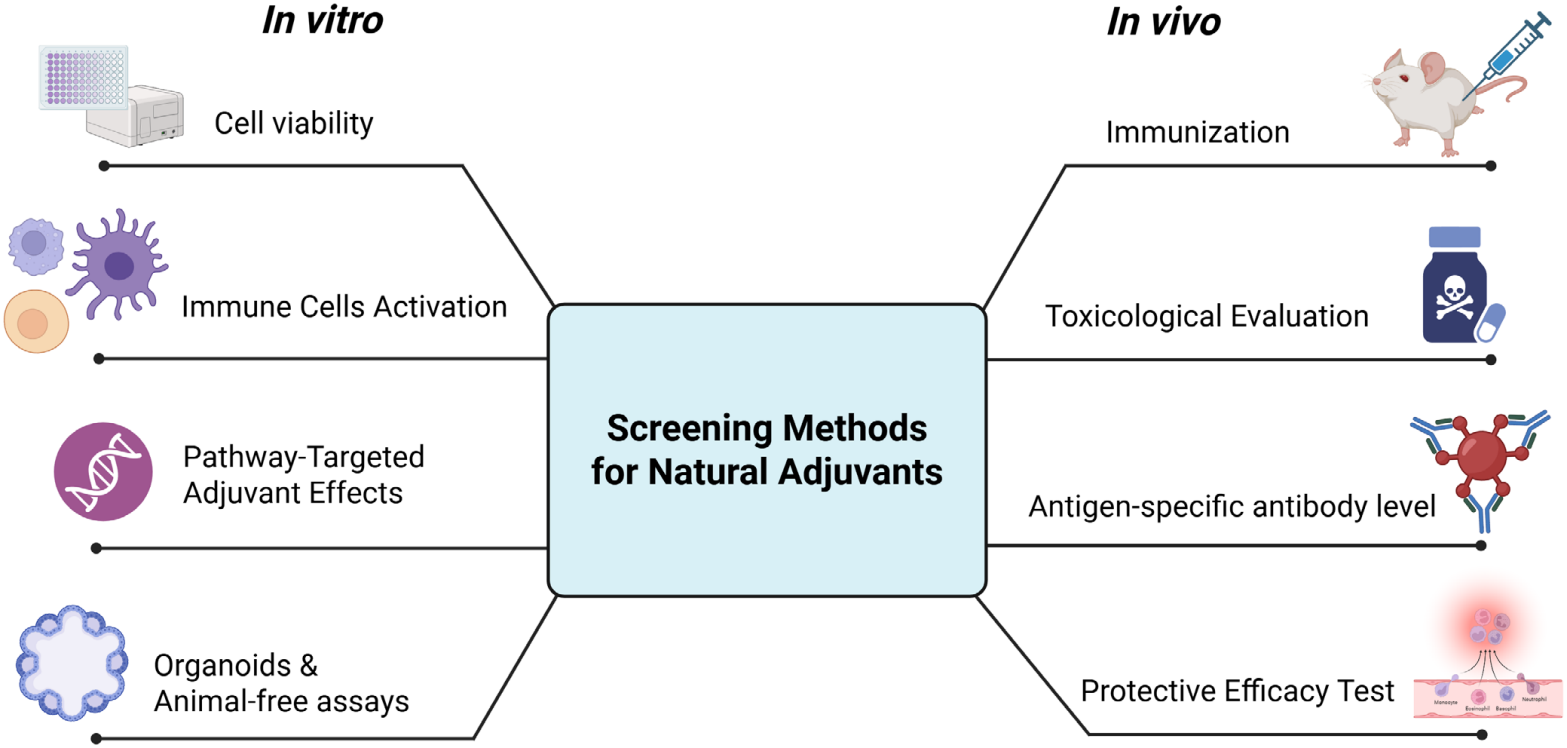
Screening methods for natural adjuvants.

The discovery and evaluation of natural products as potential vaccine adjuvants involve a combination of in vitro and in vivo methodologies. These approaches are critical for understanding the immunomodulatory properties, efficacy, and safety of candidate compounds before clinical application. A schematic overview is presented in Figure 4, illustrating the key steps in the screening pipeline.

### 4.1 In Vitro Screening Approaches

In vitro assays offer a rapid, cost‐effective, and mechanistically insightful platform to evaluate the immunostimulatory properties of natural compounds at the cellular and molecular levels. These methods serve as a critical first filter before progressing to in vivo testing.

#### 4.1.1 Innate Immune Cell Activation

Antigen‐presenting cells (APCs), such as dendritic cells (DCs) and macrophages, play a central role in coordinating innate and adaptive immune responses. Natural extracts are evaluated for their capacity to enhance the expression of co‐stimulatory surface markers, including CD80, CD86, CD40, and MHC class II, as well as to stimulate the secretion of cytokines such as TNF‐α, IL‐6, and IL‐12p70 (Ho et al. 2023). In addition to APCs, the immunostimulatory effects of natural adjuvants can also be assessed in other innate immune cells, such as natural killer (NK) cells. Key indicators of NK cell activation include surface markers like CD69 and CD107a, as well as effector molecules such as granzyme B and perforin (Le et al. 2021). Commonly used techniques for these assessments include flow cytometry, enzyme‐linked immunosorbent assay (ELISA), and real‐time quantitative PCR (qPCR).

#### 4.1.2 T Cell Proliferation and Polarization Assays

T cell co‐culture models such as dendritic cell (DC)‐T cell or macrophage‐T cell systems are widely used to evaluate the immunomodulatory capacity of natural extracts. These assays assess T cell proliferation, polarization into Th1/Th2 subsets, or differentiation into regulatory T cells (Tregs). Key readouts include cytokine production (IFN‐γ for Th1, IL‐4 for Th2). T cell proliferation is commonly measured via carboxyfluorescein succinimidyl ester (CFSE) dilution or [^3^H]‐thymidine incorporation (Whiteside 2011; Knight et al. 2011; Lyons et al. 2013). Additionally, the mixed lymphocyte reaction (MLR) is a classic functional assay used to evaluate the allostimulatory capacity of antigen‐presenting cells treated with candidate extracts (Grødeland et al. 2015).

#### 4.1.3 B Cell Activation and Antibody Response

Natural compounds are also screened for their effects on B lymphocytes, particularly in terms of promoting antibody class switching, germinal center formation, and memory B cell development. Readouts include expression of activation markers (CD69, CD27, CD38) and secretion of immunoglobulin isotypes such as IgG1, IgG2a, and IgM, assessed via ELISA or flow cytometry (Ellebedy et al. 2020; Guthmiller et al. 2025; Boonyaratanakornkit and Taylor 2019).

#### 4.1.4 Reporter Gene and Pathway Activation Assays

To dissect pathway‐specific effects, engineered reporter cell lines are utilized. These cells contain luciferase or GFP reporters under the control of signaling‐responsive promoters such as nuclear factor kappa‐light‐chain‐enhancer of activated B cells (NF‐κB), activator protein 1 (AP‐1), interferon regulatory factor 3 (IRF3), or signal transducer and activator of transcription 6 (STAT6). Such assays enable identification of compounds that target Toll‐like receptors (TLRs), retinoic acid‐inducible gene I (RIG‐I)‐like receptors, or inflammasome components (Zhang et al. 2018).

#### 4.1.5 Cytotoxicity and Biocompatibility Screening

Ensuring the safety of adjuvant candidates is critical. Cytotoxicity is assessed using assays such as 3‐(4,5‐dimethylthiazol‐2‐yl)‐2,5‐diphenyltetrazolium bromide (MTT) assay, lactate dehydrogenase (LDH) release assay, and Annexin V/propidium iodide (PI) staining (Borra et al. 2009). These assays confirm the non‐cytotoxic nature of extracts across a range of concentrations and help define therapeutic windows.

#### 4.1.6 Human‐Mimetic Models

To bridge the translational gap between conventional cell cultures and clinical outcomes, organoid models and animal‐free assays have emerged as critical frameworks for evaluating the immunogenicity and safety of natural adjuvants (Wagar 2023; Wagar et al. 2021; Chiarot and Pizza 2022). These platforms, specifically human‐derived lymphoid organoids, recapitulate the complex human immune microenvironment, enabling high‐fidelity assessment of cytokine kinetics and germinal center dynamics in vitro. Integrating these physiologically relevant systems with established in vivo protocols allows researchers to more accurately predict human reactogenicity while adhering to the 3Rs principle to minimize animal reliance.

### 4.2 In Vivo Screening Approaches

In vivo models provide the necessary physiological context to validate in vitro findings, essential for assessing systemic and mucosal efficacy, protective immunity, and toxicological safety.

#### 4.2.1 Murine Immunization Models

Murine models such as BALB/c and C57BL/6 mice are widely utilized to investigate the adjuvant potential of natural extracts co‐administered with vaccine antigens. These models enable systematic evaluation of both humoral and cellular immune responses in a controlled environment. Multiple routes of administration are available for delivering the vaccine‐adjuvant formulations, including: Intramuscular (IM) – commonly used for systemic immunity, Intranasal (IN) – relevant for mucosal immunity, Intraperitoneal (IP) – useful in exploratory and mechanistic studies, Oral and subcutaneous (SC) – depending on adjuvant solubility and intended immune compartment. The choice of route influences antigen uptake, immune cell recruitment, and the resulting immune profile. Comparative studies across delivery routes can reveal which route best enhances the adjuvant effect of a given extract. Key Immunological Endpoints.

- Humoral immunity: Measurement of antigen‐specific total IgG and subclass responses (IgG1, IgG2a) via ELISA.
- Cell‐mediated immunity: Quantification of antigen‐specific T cell responses using ELISpot assays, intracellular cytokine staining (ICS), and flow cytometry‐based phenotyping.
- Protective efficacy: Post‐challenge evaluation including viral titers in target organs (e.g., lung), clinical scoring, body weight monitoring, and survival curves.

#### 4.2.2 Cytokine and Immune Profiling

Post‐immunization, cytokine levels in serum, bronchoalveolar lavage fluid (BALF), lung, or spleen are analyzed to understand the systemic immune milieu. ELISA are commonly used to quantify cytokines such as IL‐2, IL‐4, IL‐6, IL‐10, TNF‐α, and IFN‐γ.

#### 4.2.3 Toxicological and Histopathological Evaluation

Mice are monitored for distress, weight loss, and behavioral changes to assess safety. Post‐mortem histopathology of organs (liver, spleen, kidney, lung) detects inflammation or necrosis, while hematological and biochemical panels identify systemic toxicity.

Together, these in vitro and in vivo methods provide a framework for evaluating the immunological and safety profiles of natural adjuvants. This integrative approach facilitates the selection of candidates for clinical translation.

## 5 Conclusion

Natural extracts present a valuable strategy to enhance the efficacy and safety of influenza vaccines. By activating antigen‐presenting cells, modulating cytokine responses, and promoting adaptive immunity, they improve both humoral and cellular responses. Specifically, results demonstrate that these bioactive compounds facilitate immunoglobulin isotype switching and induce robust T‐cell activation, leading to enhanced neutralizing antibody titers and superior survival rates in lethal challenge models. Many exhibits low reactogenicity and are compatible with various vaccine platforms and delivery routes. Preclinical studies support their potential for inducing long‐lasting and cross‐protective immunity. However, further research is needed to standardize formulations and confirm safety. Overall, natural extract‐based adjuvants offer a promising path toward more effective and broadly protective next‐generation influenza vaccines.

## 6 Limitations and Future Recommendations

While natural extracts are promising, limitations include significant batch‐to‐batch variability, complex chemical compositions that obscure specific active markers, and challenges in scaling high‐purity production. Future recommendations focus on using high‐throughput biological profiling for precise molecular targeting, developing synthetic analogues to ensure chemical consistency, and advancing toward human clinical trials to confirm preclinical safety and cross‐protective efficacy.

## Author Contributions

**Eun‐Ju Ko:** funding acquisition, project administration, supervision, writing – review and editing, validation, visualization. **Thi Len Ho:** conceptualization, investigation, methodology, writing – original draft, writing – review and editing, data curation, formal analysis, visualization.

## Funding

This work was supported by a National Research Foundation of Korea (NRF) grant funded by the Korean government (MSIT) (RS‐2023‐00211504) and Basic Science Research Program to the Research Institute for Basic Sciences (RIBS) of Jeju National University through the NRF, funded by the Ministry of Education (RS‐2019‐NR040080).

## Conflicts of Interest

The authors declare no conflicts of interest.

## Data Availability

The data that support the findings of this study are available from the corresponding author upon reasonable request.
